# Molecular Survey of Tularemia and Plague in Small Mammals From Iran

**DOI:** 10.3389/fcimb.2018.00215

**Published:** 2018-07-10

**Authors:** Ehsan Mostafavi, Ahmad Ghasemi, Mahdi Rohani, Leila Molaeipoor, Saber Esmaeili, Zeinolabedin Mohammadi, Ahmad Mahmoudi, Mansour Aliabadian, Anders Johansson

**Affiliations:** ^1^National Reference Laboratory for Plague, Tularemia and Q Fever, Research Centre for Emerging and Reemerging Infectious Diseases, Pasteur Institute of Iran, Kabudar Ahang, Iran; ^2^Department of Epidemiology and Biostatistics, Research Centre for Emerging and Reemerging Infectious Diseases, Pasteur Institute of Iran, Tehran, Iran; ^3^Department of Bacteriology, Faculty of Medical Sciences, Tarbiat Modares University, Tehran, Iran; ^4^Department of Microbiology, Pasteur Institute of Iran, Tehran, Iran; ^5^Department of Epidemiology, School of Public Health, Iran University of Medical Sciences, Tehran, Iran; ^6^Student Research Committee, Faculty of Public Health Branch, Iran University of Medical Sciences, Tehran, Iran; ^7^Rodentology Research Department, Applied Animal Institute, Ferdowsi University of Mashhad, Mashhad, Iran; ^8^Department of Biology, Faculty of Science, Ferdowsi University of Mashhad, Mashhad, Iran; ^9^Department of Clinical Microbiology and the Laboratory for Molecular Infection Medicine Sweden, Umeå University, Umeå, Sweden

**Keywords:** tularemia, plague, hares, rodentia, insectivora

## Abstract

**Introduction:** Plague and tularemia are zoonoses and their causative bacteria are circulating in certain regions of Iran. This study was conducted to investigate potential disease reservoirs amongst small wildlife species in different regions of Iran.

**Methods:** Rodents, insectivores and hares from 17 different provinces of the country were collected in 2014 and 2015. Samples were taken from the spleens of the animals and Real-time PCR was applied to detect nucleic acid sequences that are specific to *Francisella tularensis* and *Yersinia pestis*, respectively.

**Results:** Among 140 collected rodents, 25 distinct species were identified out of which five were the most common: *Microtus paradoxus* (21% out of 140 rodents), *Apodemus witherbyi* (12%), *Microtus irani* (11%), *Mus musculus* (11%) and *Microtus socialis* (10%). Seventeen insectivores were collected and identified as *Crocidura suaveolens* (82%) and *C. leucodon* (18%). Fifty-one hares were collected and identified as *Lepus europaeus* (57%), *Lepus tolai* (14%) and *Lepus* sp. (29%). Three out of 140 explored rodents (1.91%) were positive for *F. tularensis*, an *A. witherbyi*, a *Mus musculus domesticus*, and a *Chionomys nivalis* collected from Golestan, Khuzestan and Razavi Khorasan provinces, respectively. Two hares (3.92%) were *F. tularensis*-positive, a *L. europaeus* from Khuzestan and a *Lepus* sp. from the Sistan and Baluchistan province. None of the tested animals were positive for *Y. pestis*.

**Conclusion:** This is the first report of direct detection of *F. tularensis* in mammals of Iran and the first-time observation of the agent in a snow vole, *C. nivalis* worldwide. The results indicate that tularemia is more widespread in Iran than previously reported including the Northeast and Southwestern parts of the country. Future studies should address genetic characterization of *F. tularensis* positive DNA samples from Iran to achieve molecular subtyping and rule out assay cross-reactivity with near neighbor *Francisella* species.

## Introduction

Emerging and reemerging infectious diseases stand amongst the most challenging problems for public health entities around the world and more than half of them are zoonotic (Parhizgari et al., 2017). Changes in social, economic, environmental, and ecological factors may precipitate the conditions for the reemergence of these infectious diseases (Gupta et al., 2012). Plague and tularemia are two zoonotic diseases that are reported from Iran (Karimi et al., 1981; Esamaeili et al., 2013; Zargar et al., 2015).

Plague is a lethal zoonotic disease that historically has caused pandemics around the world. The causative agent of this disease is the bacterium *Yersinia pestis*. Plague is still endemic in certain regions of Africa, Asia, and North and South America (Dubyanskiy and Yeszhanov, 2016). Since 1990, most of human plague infections are reported from African countries (Williamson, 2016). Wild rodents and fleas on their bodies are regarded as the main reservoirs of *Y. pestis* in nature (Bitam et al., 2010); although other wild animals such as mammalian carnivores and insectivores can also play a role as the reservoir of the infection (Poland and Dennis, 1999). Infection of rodents lead to severe damages in liver and spleen, and demise in a period less than 3 days (Bevins et al., 2012). Rabbits and hares can also be infected with *Y. pestis* and may like rodents transmit the infection to humans by direct contact or indirectly via infected flea bites (Fratini et al., 2017). From 1943 to 1965, nine plague outbreaks were reported amongst humans in the western areas of Iran. In these outbreaks, rodents and hares were regarded as the main reservoirs (Shahraki et al., 2016). The last report of plague among rodents in Iran was in 1978 in the Eastern Azerbaijan province, northwest of Iran (Karimi, 1980). The studies on plague in wildlife in Iran were discontinued for a while but were restarted in 2011 and 2012 (Mostafavi and Keypour, 2017) when antibodies against *Y. pestis* were found in rodents and dogs of northwest Iran (Esamaeili et al., 2013). Over recent years, several plague outbreaks have been reported from Iran's neighboring countries such as Afghanistan, Saudi Arabia and Jordan (Saeed et al., 2005; Leslie et al., 2011).

Tularemia is caused by the bacterium *Francisella tularensis* and a vast range of rodents, hares, insectivores, ticks, flies and mosquitoes have been implicated as potential disease reservoirs (Goethert and Telford, 2009; Ulu-Kilic et al., 2013; Zargar et al., 2015). The disease is typically rapidly progressing in small mammals resulting in necrosis distributed in multiple organs including the spleen and death usually follows within a few days, alternatively, a chronic disease type with granulomas in the liver have been described (Maurin and Gyuranecz, 2016). There is incomplete knowledge of the worldwide tularemia burden among humans but generally, it is assumed to be a disease of the Northern hemisphere only. Between 2000 and 2012, 250 to 2500 human cases were reported from countries of Europe and from Turkey (Hestvik et al., 2015; Sanyaolu et al., 2016). Human mortality is generally low but disease and its symptoms including fever and fatigue may be long-lasting (Erdem et al., 2014). Outbreaks in proximity to rivers in Russia, Europe, and in Turkey points toward an important role of water in the survival of the bacterium and that aquatic rodents may serve as disease reservoirs (Sjöstedt, 2007; Kaysser et al., 2008; Clark et al., 2012). Turkey, one of Iran's neighboring countries, is accounted as an endemic region for this disease (Sahin et al., 2007). Humans can be infected via direct contact with infected animals or via bites of infected arthropods, intake of contaminated water or food, or via inhalation of *F. tularensis*-contaminated aerosols. There are several different clinical forms of disease in humans depending on the infectious route of the bacterium, all of which includes swelling of lymph nodes and may progress to septic disease (Barker and Klose, 2007). The first report of possible tularemia in Iran goes back to 1973 when antibodies against *F. tularensis* were found in domestic livestock and one hedgehog in the northwest and east of Iran, respectively (Arata et al., 1973). The first and thus far only report of a human case of tularemia was from the Marivan district, Kurdistan province of west Iran, in 1980 (Karimi et al., 1981). Studies that are more recent have shown a relatively high prevalence of *F. tularensis*-specific antibodies in the blood of humans living in the west, southeast and southwest parts of Iran. The results of these studies suggest that tularemia may be underdiagnosed in Iran and that enhanced disease surveillance would be valuable (Esmaeili et al., 2014a,b; Khoshdel et al., 2014; Zargar et al., 2015). In 2013, *F. tularensis*-specific antibodies were found in rodents in the southeast and west of Iran indicating that small mammals may be involved in disease transmission (Pourhossein et al., 2015; Mostafavi et al., 2017).

This study was conducted to evaluate the infection of small mammals including rodents, insectivores and hares with *Y. pestis* and *F. tularensis* in order to gain better information regarding the status of these agents in the wildlife of Iran.

## Materials and methods

In this cross-sectional study, small mammals were captured alive in different provinces (central, northern, southern, eastern and western) of Iran in 2014 and 2015. The captured animals were sprayed with a pesticide in order to destruct the ectoparasites on their bodies; they were then dispatched to predetermined stations and euthanized. The captured small mammals were identified according to standard identification keys (Corbet, 1978; Kryštufek et al., 2005). Splenic samples were collected for molecular studies and preserved in microtubes containing 70% alcohol before being sent to the diagnostic laboratory.

DNA extraction from tissue samples was completed using the QIAamp DNA Mini Kit. In order to trace and identify *F. tularensis* and *Y. pestis* in tissue samples, the extracted DNA was subjected to pathogen-specific Real-time PCR assays using a Rotor-Gene 6600 (Corbett Life Science). The gene targets for *Y. pestis* were the chromosomal *yih*N gene and the plasmid genes *caf1* and *pla* (Table 1). The same genes cloned into the plasmid pUC57 were used as positive control (provided by the Pasteur Institute of Iran). For *Francisella* spp. we used the IS*Ftu2* gene for a first screening step and the *fop*A gene for confirmation of the presence of *F. tularensis* as a second step. The DNA of *F. tularensis* subsp *holarctica* NCTC 10857 was used as a positive control. The DNA amplification was done in a volume of 25μl for 40 cycles after an initial denaturation at 95°C for 10 min. The cycling for *Y. pestis* was 95°C for 15 s, 58°C for 30 s, and 72°C for 30 s whereas for *F. tularensis* it was 95°C for 15 s, 58°C for 60 s, and 72°C for 60 s. As an internal control, the β-actin gene (Qiagen company) was used (Emanuel et al., 2003; Versage et al., 2003; Stewart et al., 2008; Bushon et al., 2010).

**Table 1:** Primers and probes used for detection of *F. tularensis* and *Y. pestis*.

**Agent**	**Gene target**	**Primer and probe**	**Sequence (5′ to 3′)**	**Amplicon size (bp)**	**Reference**
*F. tularensis*	IS*Ftu2* (chromosome)	ISFtu2F	TTGGTAGATCAGTTGGTAGGATAACC	97	Versage et al., 2003
		ISFtu2R	TGAGTTTTATCCTCTGACAACAATATTTC		
		Probe	FAM-AAAATCCATGCTATGACTGATGCTTTAGGTAATCCA-TAMRA		
	*fop*A (chromosome)	fopA-F	AACAATGGCACCTAGTAATATTTCTGG	87	Bushon et al., 2010
		fopA-R	CCACCAAAGAACCATGTTAAACC		
		Probe	FAM-TGGCAGAGCGGGTACTAACATGATTGGT-5-TAMRA		
*Y. pestis*	*yih*N (chromosome)	Chrom F	CGCTTTACCTTCACCAAACTGAAC	128	Stewart et al., 2008
		Chrom R	GGTTGCTGGGAACCAAAGAAGA		
		Probe	Texas Red-TAAGTACATCAATCACACCGCGACCCGCTT-BHQ-2		
	*caf1* (plasmid)	pMT1 F	CCGTTATCGCCATTGCATTATTTGG	194	
		pMT1 R	GCCAAGAGTAAGCGTACCAACAAG		
		Probe	FAM-AAGCACCACTGCAACGGCAACTCTT-BHQ-1		
	*pla* (plasmid)	pPCP1 F	ATTGGACTTGCAGGCCAGTATC	144	
		pPCP1 R	ATAACGTGAGCCGGATGTCTTC		
		Probe	FAM-AAATTCAGCGACTGGGTTCGGGCACA-BHQ-1		

All procedures performed in this study involving capturing and euthanizing of the animals were in accordance with international ethical standards. The institutional animal and human ethical committee of the Pasteur institute of Iran approved the project. Gloves, mask, face shield and gown were worn by personnel handling animals in the field and by laboratory personnel handling animal specimens. Personnel specifically trained in handling pathogenic agents performed the laboratory work. Procedures involving potentially infectious material were performed within a class II plus biological safety cabinet.

## Results

**Figure 1:**
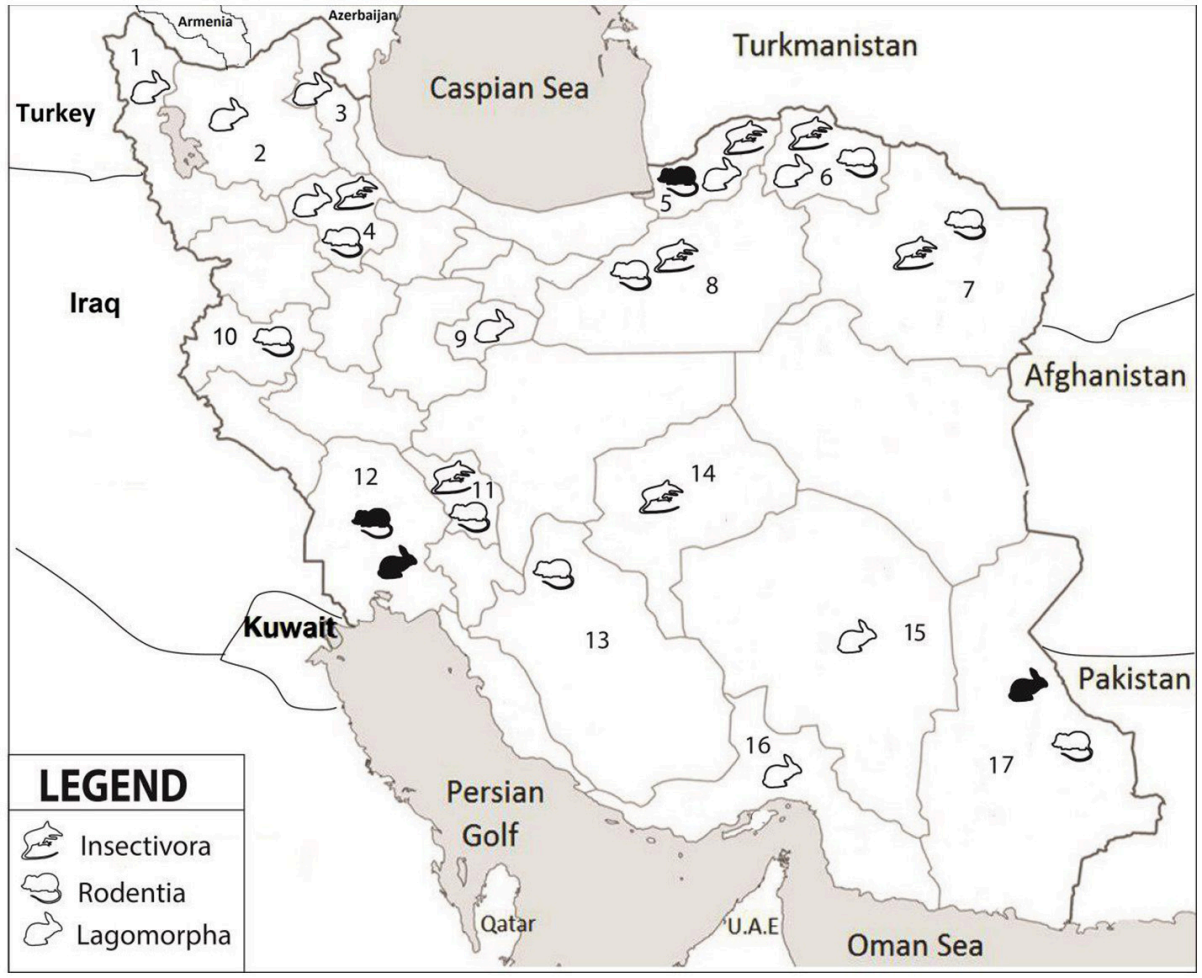
Collection locations in seventeen provinces of Iran. 1-West Azerbaijan, 2-East Azerbaijan, 3-Ardabil, 4-Zanjan, 5-Golestan, 6-North Khorasan, 7-Razavi Khorasan, 8-Semnan, 9-Qom, 10-Kermanshah, 11- Chaharmahal and Bakhtiari, 12-Khuzestan, 13-Fars, 14-Yazd, 15-Kerman, 16-Hormozgan, 17-Sistan and Baluchistan. Different groups of small mammals are indicated by symbols; white color refers to negative and black color to positive samples.

Altogether, 208 specimens from 140 rodents, 17 insectivores and 51 hares were collected from 17 different provinces (Figure 1, Table 2). The rodents were from 11 provinces including Northern Khorasan, Razavi Khorasan, Golestan, Fars, Zanjan, Chaharmahal and Bakhtiari, Semnan, Sistan and Baluchistan, Khuzestan, Kerman and Kermanshah. Of the 25 studied rodent species, *Microtus paradoxus, Apodemus witherbyi, Microtus irani, Mus musculus*, and *Microtus socialis* were most common with a frequency of 30 (21.4%), 17 (12.1%), 16 (11.4%), 15 (10.7%), and 14 (10.0%), respectively. The insectivores were identified as 14 (82.4%) *Crocidura suaveolens* and 3 (17.6%) *Crocidura leucodon*. *C. suaveolens* was collected from different parts of the country (Golestan, North Khorasan, Zanjan, Chaharmahal and Bakhtiari, while *C. leucodon* was collected from a restricted area in northern Iran (Semnan and North Khorasan). The captured hares were 29 (56.9%) *Lepus europaeus*, 7 (13.7%) *Lepus tolai* and 29 (29.4%) other *Lepus* sp. *L. europaeus* was collected in Zanjan, Khuzestan, Ardebil, Eastern Azerbaijan, Western Azerbaijan and Qom whereas the *Lepus* sp. were from Sistan and Baluchistan, Southern Khorasan, Kerman and Hormozgan. *L. tolai* was collected from the Golestan province (Table 2).

**Table 2:** Sampling locations of wild-caught rodents, insectivores, and hares.

**Animal group**	**Province (no. of collected animals)**	**Sampling site**	**Species (no.)**
Rodents and insectivores	Golestan (30)	Gorgan, Toskestan, Aliabad-e Katul	Apodemus uralensis^*^(5), *Microtus paradoxus* (10), *Mus musculus* (9), *Crocidura suaveolens* (4), *Rattus rattus*(1)*, Apodemus witherbyi* (1)
	North Khorasan (32)	Bojnord (Darkesh, Dasht, Gachranlo)	*M. paradoxus* (19)*, M. musculus* (4)*, C. suaveolens* (1), *A. witherbyi* (3), *Meriones persicus (*1), *Crocidura leucodon* (2)
	Razavi Khorasan (21)	Mashhad, Moghan, Dargaz	*Microtus transcaspicus* (*3*), Chionomys nivalis^*^(4), *A. witherbyi* (9), *M. musculus* (1), *Blanfordimys afghanus* (1), *M. persicus* (1), *C. suaveolens* (1), *M. paradoxus* (1)
	Zanjan (16)	Soltanieh, Mahneshan, Anguran	*C. suaveolens* (7), *Microtus mystacinus* (1), *Microtus qazvinensis* (5), *Mus macedonicus* (3)
	Semnan (9)	Shahmirzad, Jashlobar	*M. mystacinus* (5), *A. witherbyi* (3), *C. leucodon* (1)
	Chaharmahal and Bakhtiari (15)	Lordegan, Shahrekord	*Microtus socialis* (14), *C. suaveolens* (1)
	Kermanshah (1)	Songhor	*M. qazvinensis* (1)
	Fars (17)	Shiraz, Mamasani	*Microtus irani* (16), *A. witherbyi* (1)
	Kerman (6)	Kerman	*Microtus kermanensis* (5), *M. musculus* (1)
	Khuzestan (7)	Izeh	*Calomyscus bailwardi* (3), M. musculus domesticus^*^ (3), *M. persicus* (1)
	Sistan and Baluchistan (3)	Iran Shahr	*Jaculus branfordi* (3)
Hares	East Azerbaijan (2)	Shabestar, Mianeh	*Lepus europaeus* (2)
	West Azerbaijan (1)	Urmia	*L. europaeus* (1)
	Ardabil (5)	Parsabad Moghan, Bilesovar, Meshkinshahr, Khalkhal	*L. europaeus* (5)
	South Khorasan (4)	Khusf, Shosf	*L. europaeus* (4)
	Golestan (7)	Gorgan, Incheh Borun, Gonbad, Kordkuy	*Lepus tolai* (7)
	Zanjan (8)	Sohrein, Tarom, Zarinabad, Mahneshan, Khodabandeh, Dandi	*L. europaeus* (8)
	Kerman (4)	Anbarabad, Kahnuj	*Lepus* sp. (4)
	Khuzestan (8)	Shushtar, Ramhormoz	L. europaeus^*^ (8)
	Hormozgan (2)	Sardasht, Rudan	*Lepus* sp. (2)
	Sistan and Baluchistan (9)	Mirjaveh, Maskutan, Bazman	*Lepus* sp.^*^ (9)
	Qom (1)	Kahak	*L. europaeus*

* *F. tularensis was detected*.

Out of all wild-caught animals, three rodents and two hares were positive for *F. tularensis*. The rodents were one each of *Apodemus uralensis* (Toskestan, Golestan province), *Mus musculus domesticus* (Izeh, Khuzestan), and *Chionomys nivalis* (Zoshk village, Mashhad, Razavi Khorasan province). The hares were a *L. europaeus* from Khuzestan and a *Lepus* sp. from the Sistan and Baluchistan province. There was no detection of *Y. pestis* in any of the animals (Table 2).

## Discussion

This study is the first report of direct detection of *F. tularensis* in rodents and hares in Iran and to the best of our knowledge, the first report of infection in the rodent species *C. nivalis* worldwide. Our study also expands the known geographic distribution of *F. tularensis* in Iran. In contrast, *Y. pestis* was not detected in any of the wild-caught animals examined.

The *F. tularensis* positive samples from animals in Northeast and Southwestern parts of Iran suggests that tularemia is widely distributed in Iran. An early study performed 1969 to 1970 using sampling with serology of >4,600 small mammals and 200 cattle and sheep showed that tularemia existed in the northwest and at one location at the very east of the country (Arata et al., 1973). Over the years, additional serology findings have verified that tularemia exists also in other parts of Iran. Recent studies of rodents in the southeast and west of Iran showed the presence of antibodies against *F. tularensis* (Pourhossein et al., 2015; Mostafavi et al., 2017). In another study of humans in 2014 in the Sistan and Baluchistan province southeast of Iran, the seroprevalence of tularemia among butchers and workers of slaughterhouses was estimated to be 6.5% (Esmaeili et al., 2014b). In addition, a study on humans with risk factors for acquiring tularemia reported a 14.4% prevalence of antibodies against tularemia in Kurdistan, west Iran (Esmaeili et al., 2014a). Finally, a study of rural children in the Chaharmahal and Bakhtiari province, southwest of Iran, showed that the prevalence of antibodies to *F. tularensis* was 6% (Khoshdel et al., 2014).

Given our findings of *F. tularensis* in rodents and hares in several areas of the country including in the Northeast and the Southwest and several previous studies with findings of *F. tularensis* antibodies in humans and animals, it is noticeable that there have been no human cases or tularemia reported in Iran since 1980 (Karimi et al., 1981). Tularemia in other countries, however, is often a diagnostic challenge to the practicing doctor, especially in areas where it seldom appears (Eliasson et al., 2005). Because the clinical diagnosis is based on awareness of the disease, giving rise to a clinical suspicion, and that it may resemble other diseases, we think that tularemia may be underdiagnosed in Iran. Recent publications from Turkey illustrate that the diagnosis may easily be overlooked because it often mimics other conditions of fever such as tuberculosis and other diseases that may cause enlarged lymph nodes (Karabay et al., 2013; Erdem et al., 2014; Yildirim et al., 2014). A presence of tularemia among humans in Iran's neighboring countries such as Azerbaijan (Clark et al., 2012), Armenia (Melikjanyan et al., 1996), and Turkey (Helvaci et al., 2000; Sahin et al., 2007; Balci et al., 2014) suggests that tularemia may be underdiagnosed in Iran.

In the present study, positive samples of *F. tularensis*-infection were observed in three rodents (*A. uralensis, M. musculus domesticus*, and *C. nivalis*) and two hares (*L. europaeus* and *Lepus* sp.) in the north, southeastern and southwestern Iran. The House mouse and the pygmy field mouse (*A. uralensis*) have previously been reported as a source of *F. tularensis* (Sakiyev et al., 2013; Unal et al., 2014). However, the natural infection of the European snow vole (*C. nivalis*) with *F. tularensis* was to the best of our knowledge not reported. More than 200 species of mammals have been identified as reservoirs of tularemia and many of them are rodents. Aquatic rodents are thought to play a crucial role in disease maintenance and the connection between this bacterium and natural waters (Christova and Gladnishka, 2005; Keim et al., 2007). The significance of rodents in the transmission of this disease is also strengthened by the observation that several tularemia outbreaks in humans were following outbreaks among rodents (Mörner, 1992; Johansson et al., 2014; Rodríguez-Pastor et al., 2017). Those rodents which have been described to play a role in the transmission of tularemia throughout the world were mostly from the genera *Microtus* (vole), *Arvicola* (water vole), *Apodemus* (field mice), and *Myodes* (Red Vole) (Kaysser et al., 2008; Gyuranecz et al., 2010). The observation of *F. tularensis* in hares is also in accordance with previous knowledge of that *Lepus* (hares) are important hosts, contributing significantly to the maintenance of the natural cycle of the agent and carrying a potential to produce infection in humans (Hopla and Hopla, 1994). Our findings of *F. tularensis* in a *L. europaeus* from Khuzestan and a *Lepus* sp. from the Sistan and Baluchistan province can be put into the context that infected rabbits and hares were reported in the tularemia epidemics in 1983, 1990–1992, 2005, and 2007 in Germany (Runge et al., 2011; Stalb et al., 2017). Another example is that in 1988, one case of tularemia was reported in a hare from the genus *Lepus* in Sudan (Mörner et al., 1988).

Two main bacterial subtypes may cause tularemia, *F. tularensis* subsp. *tularensis* (type A) and *F. tularensis* subsp. *holarctica* (type B) which are traditionally connected with different disease ecologies. Type A is extensively seen in the USA and is said to be mainly connected with a terrestrial cycle of the disease (rabbits and hares are mammal hosts; arthropods including ticks serve as vectors). Type B bacteria are related with an aquatic cycle and have mostly been found in outbreaks associated with rivers, lakes, ponds and brooks (semi-aquatic rodents are mammal hosts and mosquitoes or flies serve as vectors) (Hopla, 1974; Hopla and Hopla, 1994; Helvaci et al., 2000; Ulu et al., 2011; Maurin and Gyuranecz, 2016). Because *F. tularensis* subsp*. holarctica* with connections to water has been repeatedly detected in the neighboring country Turkey (Helvaci et al., 2000; Sahin et al., 2007; Balci et al., 2014), it is probable that *F. tularensis* identified in this study would be of this subtype. In Turkey, multiple phylogenetic groups of *F. tularensis* subsp. *holarctica* have been found indicating that much genetic diversity of this subspecies exists in proximity to Iran (Özsürekci et al., 2015). The presence of *F. tularensis* subsp. *holarctica* would also fit with the observation that the European brown hare (*L. europaeus*) typically serves as host for *F. tularensis* subsp. *holarctica* as verified in studies in countries such as Germany (Runge et al., 2011; Stalb et al., 2017) and Sweden (Mörner et al., 1988). Another possibility is that the *F. tularensis* subsp. *mediasiatica* exists in Iran, as this subspecies has been found in Kazakhstan, Turkmenistan, Uzbekistan and in the Altai region of Russia (Champion et al., 2009). The current knowledge, however, suggests that subsp. *mediasiatica* is rare in these geographical areas, which are located relatively distant to Iran (Timofeev et al., 2017).

The last reported human case of plague in Iran goes back to 1965 in the Kurdistan region, west of Iran; despite identification of active foci of plague in the wildlife of western and northwestern parts of the country there have been no human cases ever since. In our study, there was no positive case of *Y. pestis* amongst the studied rodents and hares although several of the captured rodents such as *M. persicus, M. musculus*, and *Rattus rattus* were previously identified as potential reservoirs of plague (Meyer et al., 1965; Saunders and Giles, 1977; Karimi, 1980). The role of hares in the transmission of plague has also been proposed (Hopkins and Gresbrink, 1982).

In natural foci of plague in Iran, four species of the genus *Meriones* have been shown to play a crucial role in the transmission of this disease (Shahraki et al., 2016). Recent studies performed in 2011 and 2012 identified antibodies against *Y. pestis* in rodents (1%) and in dogs (3.5%) of the Kurdistan-Hamadan border (Esamaeili et al., 2013); this would imply an ongoing infection cycle in these regions. We suspect that the number of animals studied originating in the west of Iran, the area previously described to be a focus of plague, may have been too small to identify positive samples. In light of the resurgence of plague after long time periods in other countries, e.g., after 50 years in Algeria in 2008, the health system of Iran must continue the surveillance of this disease.

A limitation of our study is that bacterial culture was not performed; this was not feasible with a study design including participation of multiple provinces and demands of in-time dispatch of samples to multiple laboratories. Another limitation is that we have not subtyped the *F. tularensis*. With the current results at hand, we suggest that future studies of tularemia in Iran should include comprehensive sampling from habitats and regions where positive samples were identified in this study and differentiation of the *F. tularensis* subtype. Although we used a PCR assay targeting the *fop*A gene that according to the literature should be specific to *F. tularensis* and the closely related pathogen *F. novicida*, there is a possibility that other *Francisella* species may have cross-reacted with the primers and probes used. The *Francisella* genus contains much diversity that was only recently discovered (Emanuel et al., 2003; Challacombe et al., 2017). A recent study from the Iberian Peninsula of Europe, e.g., found a *F. hispaniensis*-like DNA sequence in the wood mouse *Apodemus sylvaticus* (de Carvalho et al., 2016).

## Conclusion

This study reports the first molecular detection of *F. tularensis* amongst rodents and hares in Iran and the first reported detection in the snow vole, *C. nivalis*, worldwide. Future studies should include additional characterization of the infectious agent. The present study and several previously conducted studies, indicate that tularemia is an endemic infectious disease of Iran. The current knowledge could be used to motivate information and educational activities among physicians and healthcare workers to increase disease awareness and diagnostic skills.

## Author contributions

EM had role in design of the study, receiving the funds, managing the study and writing and finalizing the draft of the manuscript. AG, SE, and MR had the role in laboratory testing, writing and finalizing the draft of the manuscript. AM, ZM, and MA had role in sampling, writing and finalizing the draft of the manuscript. LM and AJ had role in analysis of the data, writing and finalizing the draft of the manuscript.

### Conflict of interest statement

The authors declare that the research was conducted in the absence of any commercial or financial relationships that could be construed as a potential conflict of interest. The reviewer RP and handling Editor declared their shared affiliation.
